# Supplementary material for: Exposure to Airborne Pesticides and Its Residue in Blood Serum of Paddy Farmers in Malaysia
Source: Int J Environ Res Public Health. 2022 Jun 2;19(11):6806. doi: 10.3390/ijerph19116806 (PMC9180057; doi:10.3390/ijerph19116806)
Supplement: Supplementary file 1 [file ijerph-19-06806-s001.zip › ijerph-1683625-supplementary.pdf]

Supplementary materials

Exposure to Airborne Pesticides and its Residue in Blood Serum of Paddy Farmers in Malaysia

Siti Khairunnisaq Rudzi[1], *Yu Bin Ho[1], Eugenie Sin Sing Tan[2], Juliana Jalaludin[1], Patimah Ismail[3]

[1]Department of Environmental and Occupational Health, Faculty of Medicine and Health Sciences, Universiti Putra Malaysia, Selangor, Malaysia

[2]School of Healthy Aging, Aesthetic and Regenerative Medicine, Faculty of Medicine and Health Sciences, UCSI University, Kuala Lumpur, Malaysia

[3]Department of Biomedical Science, Faculty of Medicine and Health Sciences, Universiti Putra Malaysia, Selangor, Malaysia

***Corresponding author**

Yu Bin Ho, PhD

Department of Environmental and Occupational Health, Faculty of Medicine and Health Sciences, Universiti Putra Malaysia, Selangor, Malaysia

E-mail: yubin@upm.edu.my

Tel.: +603-86092955

Supplementary Material S1 Chemicals and standards

High performance liquid chromatography (HPLC) grade methanol, HPLC grade acetone, HPLC grade dichloromethane (DCM), HPLC grade acetonitrile were purchased from Fisher Scientific (UK). Hydrochloric acid (HCl) (37%), formic acid (90%), and magnesium sulphate ($MgSO_4$) were purchased from R&M Chemicals (UK), sodium chloride (NaCl) for analysis was purchased from Merck (USA), while ammonium formate solution was purchased from Sigma Aldrich (Germany). Ultrapure water was produced by Millipore Milli-Q Gradient A10 (France). Native standards which were chlorantraniliprole (99.5%), azoxystrobin (99.5%), imidacloprid (99.4%), pymetrozine (99.0%), isoprothiolane (99.0%), buprofezin (99.0%), propiconazole (99.0%), trifloxystribin (99.0%), fipronil (99.0%), difenoconazole (98.7%), pretilachlor (98.7%), tricyclazole (98.5%), tebuconazole (98.5%), and internal standard (IS) imidacloprid-d_4 (98.0%) were purchased from Dr. Ehrenstorfer (Germany).

Table S1 UHPLC-MS/MS method conditions

Parameters	Conditions
Column	Eclipse Plus C18 column (2.1mm×50mm I.D., 1.8µm particle size) (Agilent, USA)
Mobile phase	A (aqueous): ultrapure water with 0.1% formic acid and 5mM ammonium formate<br>B (solvent): methanol with 0.1% formic acid and 5mM ammonium formate
Injection volume (µL)	Air sample: 2 µL<br>Blood sample: 10 µL
Flow rate (mL min^{-1})	0.5
Run time (minute)	20 minutes per injection
Column temperature (°C)	40
Mode	Electrospray ionization (ESI) positive and negative
Capillary voltage (V)	3500
Gas temperature (°C)	220
Gas flow (L min^{-1})	11
Nebulizer (psi)	30

Table S2 Gradient condition of mobile phase

	Time (minutes)	A%	B%	Flow rate (mL min^{-1})
1	0	94.00	6.00	0.5
2	15.00	2.00	98.00	0.5
3	18.00	2.00	98.00	0.5
4	18.01	94.00	6.00	0.5
5	20.00	94.00	6.00	0.5

Table S3 Climatological information

Climatological parameters	Median (IQR)	
	Farmers (n=85)	Non-farmers (n=85)
Wind speed (ms^{-1})	0.30 (0.10-0.60)	0.20 (0.10-0.30)
Temperature (ºC)	28.90 (27.25-31.00)	29.10 (25.20-30.90)
Relative humidity (%)	90.00 (77.00-96.00)	66.00 (61.50-68.50)

Table S4 Information of PPE used by paddy farmers and personal hygiene practices(n=85)

Types of PPE	Frequency, n (%)
Boot/ footwear	67 (78.8)
Gloves	23 (27.1)
Full body coverall	2 (2.4)
Waterproof pants	4 (4.7)
Waterproof apron	4 (4.7)
Long sleeved shirt	85 (100.0)
Long pants	85 (100.0)
Respirator	5 (5.9)
Old cloth as face mask	79 (92.9)
Goggle	8 (9.4)
Cap/headcover	70 (82.4)
Without any PPE	1 (1.2)
**Personal hygiene**	**Frequency, n (%)**
Wash up immediately after spraying	85 (100.0)
Change clothes after spraying	85 (100.0)

Table S5 Reasons of not using proper PPE among farmers (n=85)

Reasons	Frequency, n (%)
Uncomfortable	71 (83.5)
Do not know how to use	3 (3.5)
Do not provided	0 (0)
Expensive cost	5 (5.9)
Unsure the importance of using proper PPE	6 (7.1)

Table S6 Self-reported health symptoms by farmers (n=85) and non-farmers (n=85)

Health symptoms	Frequency, n (%)	
	Farmers	Non-farmers
Breathing difficulty	20 (23.5)	2 (2.4)
Chest pain	26 (30.6)	1 (1.2)
Cough	30 (35.3)	28 (32.9)
Phlegm	14 (16.5)	4 (4.7)
Wheezing	13 (15.3)	0 (0)
Sore throat	19 (22.4)	14 (16.5)
Nausea	40 (47.1)	5 (5.9)
Vomiting	16 (18.8)	3 (3.5)
Dizziness	42 (49.4)	4 (4.7)

Table S7 Association of pesticides concentrations in personal air samples with health symptoms among farmers (n=85)

Concentration of pesticides in personal air	Breathing difficulties		Chest pain		Cough		Phlegm		Wheezing	
	p-value	Crude OR (95% CI)	*p*-value	Crude OR (95% CI)	*p*-value	Crude OR (95% CI)	*p*-value	Crude OR (95% CI)	*p*-value	Crude OR (95% CI)
Azoxystrobin	0.30	0.98 (0.95, 1.02)	0.20	0.98 (0.94, 1.01)	0.22	0.98 (0.95, 1.01)	0.92	1.00 (0.94, 1.08)	0.22	0.98 (0.95, 1.01)
Buprofezin	0.93	1.00 (0.97, 1.03)	0.74	1.00 (0.98, 1.02)	0.23	1.02 (0.99, 1.06)	0.33	1.02 (0.98, 1.06)	0.92	1.00 (0.98, 1.02)
Chlorantraniliprole	0.43	1.00 (0.99, 1.00)	0.62	1.00 (0.99, 1.01)	0.20	1.01 (1.00, 1.01)	0.20	1.01 (0.99, 1.04)	0.84	1.00 (0.99, 1.01)
Difenoconazole	0.59	0.99 (0.96, 1.02)	0.97	1.00 (0.98, 1.02)	0.15	1.05 (0.98, 1.12)	0.23	1.05 (0.97, 1.14)	0.83	1.00 (0.97, 1.03)
Fipronil	0.61	1.00 (0.98, 1.01)	0.45	0.99 (0.98, 1.01)	0.14	1.02 (0.99, 1.04)	0.39	1.01 (0.99, 1.03)	0.30	1.02 (0.98, 1.06)
Imidacloprid	0.15	0.98 (0.96, 1.01)	0.97	1.00 (0.98, 1.03)	0.24	1.03 (0.98, 1.09)	0.75	1.01 (0.97, 1.05)	0.82	1.00 (0.97, 1.04)
Isoprothiolane	0.61	1.00 (0.99, 1.02)	0.50	1.00 (0.99, 1.01)	0.98	1.00 (0.99, 1.01)	0.65	1.00 (0.98, 1.01)	0.32	0.99 (0.98, 1.02)
Pretilachlor	0.10	0.99 (0.98, 1.00)	0.93	1.00 (0.99, 1.01)	0.61	1.00 (0.98, 1.01)	0.59	1.00 (0.99, 1.02)	0.44	0.99 (0.98, 1.03)
Propiconazole	0.18	1.01 (0.99, 1.04)	0.79	1.00 (0.99, 1.01)	0.10	0.99 (0.98, 1.00)	0.13	0.99 (0.98, 1.00)	0.81	1.00 (0.98, 1.01)
Pymetrozine	0.31	1.00 (0.99, 1.00)	0.39	1.00 (0.99, 1.01)	0.69	1.00 (0.99, 1.01)	0.24	0.99 (0.98, 1.01)	0.24	0.99 (0.98, 1.00)
Tebuconazole	0.87	1.00 (0.98, 1.03)	0.37	1.01 (0.99, 1.04)	0.43	1.01 (0.99, 1.04)	0.72	1.01 (0.97, 1.05)	0.81	1.00 (0.98, 1.03)
Tricyclazole	0.57	1.02 (0.95, 1.09)	0.92	1.00 (0.95, 1.06)	0.35	0.97 (0.92, 1.03)	0.28	0.96 (0.89, 1.04)	0.88	1.00 (0.95, 1.05)
Trifloxystrobin	0.80	1.00 (0.99, 1.01)	0.77	1.00 (0.99, 1.01)	0.21	0.99 (0.97, 1.01)	0.37	0.99 (0.98, 1.01)	0.81	1.00 (0.99, 1.02)

Table S7 Continue…

Concentration of pesticides in personal air	Sore throat		Nausea		Vomiting		Dizziness	
	p-value	Crude OR (95% CI)	*p*-value	Crude OR (95% CI)	*p*-value	Crude OR (95% CI)	*p*-value	Crude OR (95% CI)
Azoxystrobin	0.59	0.99 (0.96, 1.03)	0.38	1.02 (0.98, 1.06)	0.55	1.02 (0.95, 1.10)	0.48	0.99 (0.96, 1.02)
Buprofezin	0.89	1.00 (0.97, 1.03)	0.52	1.01 (0.99, 1.02)	0.33	0.99 (0.97, 1.01)	0.49	1.01 (0.99, 1.02)
Chlorantraniliprole	0.13	1.06 (0.98, 1.13)	0.61	1.00 (1.00, 1.01)	0.42	1.00 (0.99, 1.02)	0.90	1.00 (0.99, 1.01)
Difenoconazole	0.54	1.01 (0.98, 1.01)	0.34	1.00 (0.97, 1.01)	0.10	0.98 (0.96, 1.00)	0.73	1.00 (0.98, 1.02)
Fipronil	0.80	1.00 (0.98, 1.03)	0.38	0.99 (0.98, 1.01)	0.63	1.01 (0.99, 1.02)	0.80	1.00 (0.99, 1.02)
Imidacloprid	0.62	1.01 (0.97, 1.05)	0.13	0.98 (0.95, 1.01)	0.06	0.97 (0.95, 1.00)	0.29	0.99 (0.96, 1.01)
Isoprothiolane	0.13	0.99 (0.97, 1.00)	0.13	1.01 (1.00, 1.02)	0.33	1.01 (0.99, 1.03)	0.08	1.01 (1.00, 1.02)
Pretilachlor	0.42	0.99 (0.98, 1.01)	0.56	1.00 (0.99, 1.01)	0.35	0.99 (0.98, 1.01)	0.58	1.00 (0.99, 1.01)
Propiconazole	0.29	0.99 (0.98, 1.01)	0.15	1.01 (1.00, 1.02)	0.77	1.00 (0.99, 1.02)	0.63	1.00 (0.99, 1.01)
Pymetrozine	0.52	1.00 (0.99, 1.01)	0.19	0.99 (0.98, 1.00)	0.07	0.99 (0.98, 1.00)	0.13	0.99 (0.97, 1.00)
Tebuconazole	0.12	0.98 (0.95, 1.01)	0.61	0.99 (0.97, 1.02)	0.44	0.99 (0.96, 1.02)	0.23	1.01 (0.99, 1.04)
Tricyclazole	0.19	1.06 (0.97, 1.15)	0.82	1.01 (0.96, 1.05)	0.42	1.02 (0.97, 1.08)	0.18	0.97 (0.92, 1.02)
Trifloxystrobin	0.92	1.00 (0.98, 1.02)	0.55	1.00 (0.98, 1.01)	0.49	0.99 (0.98, 1.01)	0.54	1.00 (0.98, 1.01)

Table S8 Association of pesticides concentrations in blood serum samples and confounder (PPE) with health symptoms among farmers (n=85)

Concentration of pesticides in blood serum	Breathing difficulties				Chest pain				Cough			
	p-value	Crude OR (95% CI)	*p*-value	Adjusted OR (95% CI)	*p*-value	Crude OR (95% CI)	*p*-value	Adjusted OR (95% CI)	*p*-value	Crude OR (95% CI)	*p*-value	Adjusted OR (95% CI)
Azoxystrobin	0.92	1.00 (0.99, 1.01)	0.83	1.00 (0.99, 1.01)	0.65	1.00 (0.99, 1.01)	0.73	1.00 (0.99, 1.01)	0.19	0.99 (0.98, 1.00)	0.16	0.99 (0.98, 1.00)
Buprofezin	0.24	1.01 (0.99, 1.04)	0.24	1.01 (0.99, 1.04)	0.95	1.00 (0.99, 1.01)	0.89	1.00 (0.99, 1.01)	0.82	1.00 (0.97, 1.02)	0.79	1.00 (0.97, 1.02)
Chlorantraniliprole	0.38	1.01 (0.99, 1.02)	0.31	1.01 (0.99, 1.02)	0.82	1.00 (0.99, 1.01)	0.95	1.00 (0.99, 1.01)	0.66	1.00 (0.99, 1.01)	0.55	1.00 (0.99, 1.02)
Difenoconazole	0.82	1.00 (0.98, 1.01)	0.67	1.00 (0.98, 1.01)	0.98	1.00 (0.98, 1.02)	0.87	1.00 (0.98, 1.02)	0.24	1.11 (0.93, 1.31)	0.25	1.11 (0.93, 1.33)
Fipronil	0.77	1.00, 0.98, 1.02)	0.63	1.00, 0.98, 1.01)	0.28	0.99 (0.98, 1.01)	0.21	0.99 (0.97, 1.01)	0.45	0.99 (0.98, 1.01)	0.37	0.99 (0.97, 1.01)
Imidacloprid	0.10	0.99 (0.98, 1.00)	0.07	0.99 (0.97, 1.00)	0.26	0.99 (0.98, 1.01)	0.20	0.99 (0.98, 1.00)	0.22	1.02 (0.99, 1.05)	0.25	1.02 (0.99, 1.05)
Isoprothiolane	0.35	0.99 (0.98, 1.01)	0.32	0.99 (0.98, 1.01)	0.63	1.00 (0.98, 1.01)	0.59	1.00 (0.98, 1.01)	0.17	1.02 (0.99, 1.04)	0.18	1.02 (0.99, 1.04)
Pretilachlor	0.98	1.00 (0.98, 1.02)	0.89	1.00 (0.98, 1.02)	0.35	1.01 (0.99, 1.04)	0.47	1.01 (0.99, 1.03)	0.11	0.98 (0.95, 1.01)	0.08	0.97 (0.95, 1.00)
Propiconazole	0.39	1.01 (0.98, 1.04)	0.42	1.01 (0.98, 1.04)	0.55	1.01 (0.99, 1.03)	0.59	1.01 (0.99, 1.03)	0.14	0.98 (0.96, 1.01)	0.13	0.98 (0.96, 1.01)
Pymetrozine	0.47	1.01 (0.99, 1.03)	0.38	1.01 (0.99, 1.03)	0.57	1.01 (0.99, 1.02)	0.46	1.01 (0.99, 1.02)	0.64	1.00 (0.99, 1.02)	0.48	1.01 (0.99, 1.03)
Tebuconazole	0.12	0.99 (0.98, 1.00)	0.11	0.99 (0.98, 1.00)	0.27	0.99 (0.98, 1.01)	0.25	0.99 (0.98, 1.01)	0.27	1.01 (0.99, 1.03)	0.27	1.01 (0.99, 1.03)
Tricyclazole	0.52	1.02 (0.96, 1.09)	0.48	1.02 (0.96, 1.09)	0.45	1.02 (0.97, 1.08)	0.41	1.02 (0.97, 1.08)	0.95	1.00 (0.92, 1.08)	0.95	1.00 (0.92, 1.08)
Trifloxystrobin	0.92	1.00 (0.98, 1.02)	0.73	1.00 (0.99, 1.02)	0.61	1.00 (0.98, 1.01)	0.88	1.00 (0.98, 1.02)	0.21	1.03 (0.98, 1.08)	0.17	1.04 (0.98, 1.09)

Table S8 Continue…

Concentration of pesticides in blood serum	Phlegm				Wheezing				Sore throat			
	p-value	Crude OR (95% CI)	*p*-value	Adjusted OR (95% CI)	*p*-value	Crude OR (95% CI)	*p*-value	Adjusted OR (95% CI)	*p*-value	Crude OR (95% CI)	*p*-value	Adjusted OR (95% CI)
Azoxystrobin	0.21	0.99 (0.98, 1.01)	0.17	0.99 (0.98, 1.00)	0.71	1.00 (0.99, 1.01)	0.66	1.00 (0.99, 1.01)	0.50	1.00 (0.99, 1.01)	0.35	0.99 (0.98, 1.01)
Buprofezin	0.73	1.00 (0.97, 1.02)	0.72	1.00 (0.97, 1.03)	0.69	1.00 (0.99, 1.02)	0.68	1.00 (0.99, 1.02)	0.42	1.01 (0.99, 1.03)	0.36	1.01 (0.99, 1.03)
Chlorantraniliprole	0.14	1.02 (0.99, 1.06)	0.13	1.02 (0.99, 1.06)	0.62	1.00 (0.99, 1.01)	0.66	1.00 (0.99, 1.01)	0.11	1.02 (1.00, 1.04)	0.60	1.02 (1.00, 1.05)
Difenoconazole	0.35	1.07 (0.93, 1.24)	0.38	1.07 (0.92, 1.23)	0.33	0.99 (0.98, 1.01)	0.30	0.99 (0.98, 1.01)	0.95	1.00 (0.98, 1.02)	0.58	1.00 (0.98, 1.01)
Fipronil	0.72	1.00 (0.98, 1.02)	0.56	0.99 (0.98, 1.01)	0.26	1.02 (0.98, 1.06)	0.27	1.02 (0.98, 1.06)	0.74	1.00 (0.98, 1.02)	0.41	0.99 (0.97, 1.01)
Imidacloprid	0.86	1.00 (0.98, 1.02)	0.95	1.00 (0.98, 1.02)	0.96	1.00 (0.98, 1.02)	0.99	1.00 (0.98, 1.02)	0.95	1.00 (0.98, 1.02)	0.83	1.00 (0.98, 1.02)
Isoprothiolane	0.45	1.01 (0.99, 1.03)	0.55	1.01 (0.99, 1.03)	0.36	0.99 (0.97, 1.01)	0.33	0.99 (0.97, 1.01)	0.38	0.99 (0.98, 1.01)	0.29	0.99 (0.98, 1.01)
Pretilachlor	0.20	0.98 (0.96, 1.01)	0.13	0.98 (0.95, 1.01)	0.13	0.98 (0.95, 1.01)	0.12	0.98 (0.95, 1.01)	0.20	0.99 (0.97, 1.01)	0.10	0.98 (0.96, 1.00)
Propiconazole	0.46	0.99 (0.97, 1.01)	0.38	0.99 (0.97, 1.01)	0.98	1.00 (0.97, 1.03)	0.99	1.00 (0.97, 1.03)	0.13	0.99 (0.97, 1.00)	0.09	0.98 (0.96, 1.00)
Pymetrozine	0.41	0.99 (0.98, 1.01)	0.68	1.00 (0.98, 1.02)	0.90	1.00 (0.98, 1.02)	0.98	1.00 (0.98, 1.02)	0.57	1.01 (0.99, 1.03)	0.30	1.01 (0.99, 1.03)
Tebuconazole	0.60	1.01 (0.99, 1.03)	0.57	1.01 (0.98, 1.03)	0.51	0.99 (0.98, 1.01)	0.51	0.99 (0.98, 1.01)	0.42	0.99 (0.98, 1.01)	0.31	0.99 (0.98, 1.01)
Tricyclazole	0.47	0.97 (0.89, 1.06)	0.48	0.97 (0.88, 1.06)	0.86	1.01 (0.93, 1.09)	0.86	1.01 (0.93, 1.09)	0.52	1.02 (0.96, 1.08)	0.38	1.03 (0.97, 1.09)
Trifloxystrobin	0.36	1.02 (0.98, 1.06)	0.26	1.02 (0.98, 1.07)	0.57	1.02 (0.96, 1.08)	0.56	1.02 (0.96, 1.08)	0.93	1.00 (0.98, 1.02)	0.58	1.01 (0.99, 1.03)

Table S8 Continue…

Concentration of pesticides in blood serum	Nausea				Vomiting				Dizziness			
	p-value	Crude OR (95% CI)	*p*-value	Adjusted OR (95% CI)	*p*-value	Crude OR (95% CI)	*p*-value	Adjusted OR (95% CI)	*p*-value	Crude OR (95% CI)	*p*-value	Adjusted OR (95% CI)
Azoxystrobin	0.22	1.01 (1.00, 1.02)	0.24	1.01 (1.00, 1.02)	0.66	1.00 (0.99, 1.02)	0.71	1.00 (0.99, 1.02)	0.66	1.00 (0.99, 1.01)	0.72	1.00 (0.99, 1.01)
Buprofezin	0.75	1.00 (0.99, 1.01)	0.77	1.00 (0.99, 1.01)	0.21	0.99 (0.98, 1.01)	0.23	0.99 (0.98, 1.01)	0.20	0.99 (0.98, 1.01)	0.22	0.99 (0.98, 1.01)
Chlorantraniliprole	0.21	1.01 (1.00, 1.02)	0.19	1.01 (1.00, 1.02)	0.20	1.01 (0.99, 1.03)	0.18	1.01 (0.99, 1.03)	0.35	1.01 (1.00, 1.01)	0.29	1.01 (1.00, 1.02)
Difenoconazole	0.53	1.00 (0.98, 1.01)	0.51	1.00 (0.98, 1.01)	0.12	0.99 (0.97, 1.00)	0.08	0.99 (0.97, 1.00)	0.33	1.01 (0.99, 1.02)	0.40	1.01 (0.99, 1.02)
Fipronil	0.94	1.00 (0.98, 1.02)	0.89	1.00 (0.98, 1.02)	0.56	1.01 (0.98, 1.04)	0.72	1.01 (0.98, 1.03)	0.43	1.01 (0.99, 1.02)	0.52	1.01 (0.99, 1.02)
Imidacloprid	0.18	0.99 (0.98, 1.01)	0.17	0.99 (0.98, 1.00)	0.09	1.00 0.99, 1.00)	0.07	0.99, 0.97, 1.00)	0.68	1.00 (0.99, 1.01)	0.59	1.00 (0.98, 1.01)
Isoprothiolane	0.39	1.01 (0.99, 1.02)	0.40	1.01 (0.99, 1.02)	0.82	1.00 (0.98, 1.01)	0.82	1.00 (0.98, 1.01)	0.23	1.01 (1.00, 1.02)	0.24	1.01 (0.99, 1.02)
Pretilachlor	0.84	1.00 (0.98, 1.02)	0.80	1.00 (0.98, 1.02)	0.85	1.00 (0.98, 1.03)	0.98	1.00 (0.98, 1.02)	0.62	1.01 (0.99, 1.02)	0.74	1.00 (0.99, 1.02)
Propiconazole	0.16	1.02 (0.99, 1.04)	0.16	1.02 (0.99, 1.04)	0.71	1.01 (0.98, 1.03)	0.76	1.00 (0.98, 1.03)	0.37	1.01 (0.99, 1.03)	0.39	1.01 (0.99, 1.03)
Pymetrozine	0.08	0.99 (0.97, 1.00)	0.09	0.99 (0.97, 1.00)	0.07	0.99 (0.97, 1.00)	0.11	0.99 (0.97, 1.00)	0.09	0.99 (0.97, 1.00)	0.11	0.99 (0.97, 1.00)
Tebuconazole	0.14	0.99 (0.97, 1.00)	0.14	0.99 (0.97, 1.01)	0.15	0.99 (0.98, 1.00)	0.13	0.99 (0.97, 1.00)	0.36	1.01 (0.99, 1.02)	0.38	1.01 (0.99, 1.02)
Tricyclazole	0.78	1.01 (0.96, 1.05)	0.78	1.01 (0.96, 1.05)	0.85	1.01 (0.95, 1.07)	0.75	1.01 (0.95, 1.07)	0.09	0.95 (0.89, 1.01)	0.10	0.95 (0.90, 1.01)
Trifloxystrobin	0.44	0.99 (0.97, 1.01)	0.52	0.99 (0.97, 1.02)	0.54	0.99 (0.97, 1.02)	0.77	1.00 (0.97, 1.02)	0.43	0.99 (0.98, 1.01)	0.58	1.00 (0.98, 1.01)
